# Soft X-ray Absorption Spectroscopic Investigation of Li(Ni_0.8_Co_0.1_Mn_0.1_)O_2_ Cathode Materials

**DOI:** 10.3390/nano10040759

**Published:** 2020-04-15

**Authors:** Jitendra Pal Singh, Jae Yeon Park, Keun Hwa Chae, Docheon Ahn, Sangsul Lee

**Affiliations:** 1 Pohang Accelerator Laboratory, Pohang University of Science and Technology, Pohang 37673, Korea; jitendra2029@postech.ac.kr; 2 Radiation Equipment Research Division, Korea Atomic Energy Research Institute, Jeongup 56212, Korea; jaeyeon@kaeri.re.kr; 3 Advanced Analysis Center, Korea Institute of Science and Technology, Seoul 02792, Korea; khchae@kist.re.kr

**Keywords:** cathode materials, orbital symmetry states, metal–oxygen hybridization

## Abstract

Herein, we report the soft X-ray absorption spectroscopic investigation for Li(Ni_0.8_Co_0.1_Mn_0.1_)O_2_ cathode material during charging and discharging. These measurements were carried out at the Mn *L*-, Co *L*-, and Ni *L*-edges during various stages of charging and discharging. Both the Mn and Co *L*-edge spectroscopic measurements reflect the invariance in the oxidation states of Mn and Co ions. The Ni *L*-edge measurements show the modification of the oxidation state of Ni ions during the charging and discharging process. These studies show that *e_g_* states are affected dominantly in the case of Ni ions during the charging and discharging process. The O *K*-edge measurements reflect modulation of metal–oxygen hybridization as envisaged from the area-ratio variation of spectral features corresponding to *t_2g_* and *e_g_* states.

## 1. Introduction

Rechargeable lithium batteries with layered oxide cathode materials have rapidly risen to prominence as fundamental devices for green and sustainable energy [1,2,3]. Thus, efforts to understand electrochemistry to improve these batteries’ performances in terms of commercial development for electric vehicles [4] and large-scale grid storage applications [5] are underway. Charging capacity, thermal stability, and capacity fading are certain factors that determine a battery’s performance [6,7], leaving ample scope for researchers to investigate. These efforts are reflected in the recent structure and electronic/atomic structural investigation of cathode materials by numerous techniques in the context of battery performance [8,9,10]. The metal ions of these materials undergo transformations in terms of oxidation state and local structural order during typical battery operation [11,12]. This affects the nature of metal–oxygen hybridization. It has been reported that these factors are influenced by the orbital symmetry states of materials’ constituent ions [13,14]. Thus, investigations related to these orbital energy levels using an appropriate technique can provide atomic level insights during the charging and discharging of batteries.

X-ray absorption spectroscopic (XAS) measurements based on soft X-rays can probe the *L*-edge of transition metals, which are important constituents of layered oxide cathode materials. These measurements, termed near-edge X-ray absorption fine structures (NEXAFS), depict orbital symmetry states associated with the oxidation of metal ions [15,16]. The O *K*-edge of these layered oxide materials can also be successfully investigated with soft X-rays. The O *K*-edge NEXAFS measurements infer the symmetry states associated with metal–oxygen interactions in these materials [17,18]. Thus, soft XAS measurements can give a complete account of underlying phenomena due to the orbital symmetry states in the oxide cathode material during charging and discharging. To depict these phenomena, the Ni-rich cathode material, Li(Ni_0.8_Co_0.1_Mn_0.1_)O_2_, is selected for the present investigation and denoted as NCM811. NCM811 is a well-known layered oxide cathode material and preferred for Li rechargeable batteries due to its high capacity [19,20]. Thus, this work investigates NCM811 cathode materials during charging and discharging using soft XAS.

## 2. Experimental Details 

X-ray diffraction (XRD) experiments prior to electrochemical testing of cathode material were performed with the 9B high-resolution powder diffraction (HRPD) beamline of the Pohang accelerator laboratory (PAL) [21]. Pristine cathode material was also investigated using X-ray absorption near-edge spectroscopy (XANES) imaging measurements to reveal the local electronic structure. These measurements at the Mn *K-*, Co *K-*, and Ni *K*-edge were performed with the 7C X-ray nano imaging (XNI) beamline of the PAL. This beamline utilizes zone plate-based transmission X-ray nanoscopy, which is a kind of transmission X-ray microscopy (TXM), for image formation. A zone plate of diameter 150 µm and 40 nm outermost zone width was used for measurements. This arrangement gave a spatial resolution of 40 nm and a field of view (FOV) of around 50 µm. The X-ray absorption images based on TXM were captured at various energies within a 1 eV interval in the range −20 to 80 eV from the main edge energies of each respective element. The XANES spectrum was extracted from these X-ray absorption images [22]. 

Soft XAS measurements of this cathode material at various charging and discharging states were performed with the 10D XAS-KIST beamline of the same laboratory in total electron yield (TEY) mode. The grating with 1100 grooves/mm was used to measure spectra at the Mn *L-*, Co *L-*, Ni *L-*, and O *K*-edges. The obtained spectra were background-subtracted and normalized with respect to the post-edge height [23]. 

## 3. Results and Discussion

**Figure 1:**
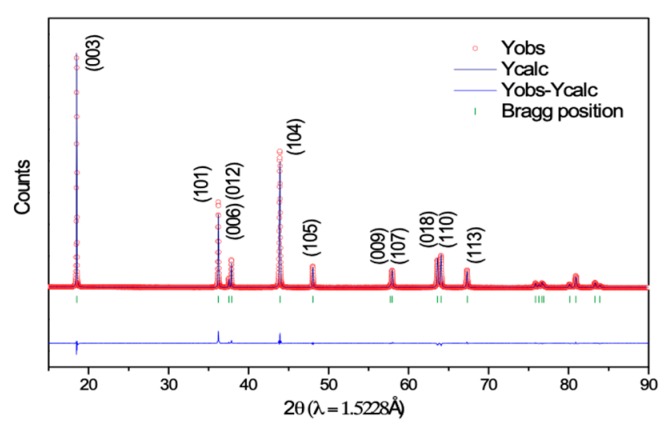
Rietveld refinement of the synchrotron high-resolution powder diffraction patterns of NCM811 cathode material prior to electrochemical testing.

Figure 1 shows the Rietveld refinement of the synchrotron high-resolution powder diffraction patterns of the NMC811 cathode material. The diffraction peaks in the XRD patterns are associated with rhombohedral R3m space group with Li-ion on the 3a site, transition metal ions on the 3b site, and oxygen ion on the 6c site [24]. The Rietveld refinement procedure is adopted to estimate the structural parameter for this material. Table 1 collates the structural and refined parameters. The refined values of the lattice parameter “a” and “c” are 2.871 and 14.195 Å, respectively. The unit cell volume is 101.34 Å^3^. The transition metal oxide (TM–O) and Li–O bond distances are 1.978 and 2.0978 Å, respectively. These parameters are similar to those reported for this composition of NMC cathode material [25]. 

In this structure, almost 2.52% of Ni ions occupy the Li-ions site (Table 1). This kind of effect is common in NCM cathode materials and associated with the almost similar size of Li and Ni ions [26]. 

**Figure 2:**
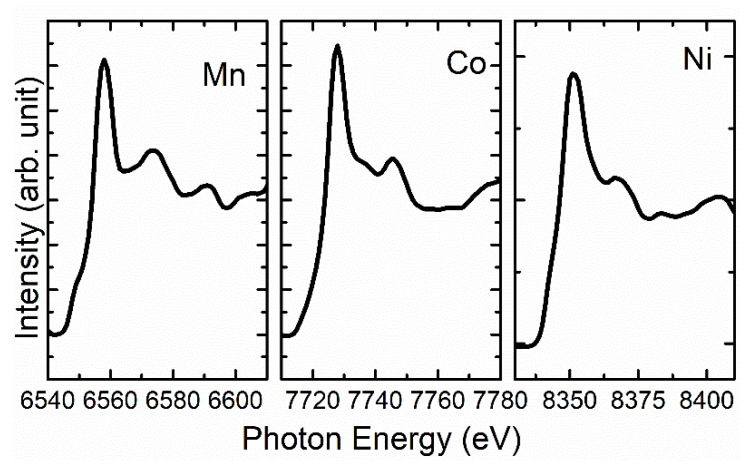
X-ray absorption near-edge spectroscopy (XANES) spectra at the Mn, Co, and Ni *K*-edges extracted (bulk mode) from transmission X-ray microscopy (TXM) images for NMC811 cathode material.

The chemical state of the constituent ions of the NCM811 cathode material was investigated using XANES-imaging measurements. Figure 2 shows the XANES spectra of the NCM811 cathode material extracted from the TXM images. In Figure 2, these spectra are shown at the Ni K-edge, Mn K-edge, and Co K-edge extracted from the original TXM images. These spectra exhibit that the main edge energy of Mn, Co, and Ni ions exist at the energy of 6.552 ± 1, 7.721 ± 1, and 8.342 ± 1 eV, respectively, in this cathode material. These values are higher than those corresponding to metal edges for each ion showing a higher oxidation state of Mn (6.539 eV), Co (7.709 eV), and Ni (8.333 eV) ions in cathode material [27]. The value of the Mn K-edge energy is similar to that of MnO_2_, revealing the 4+ oxidation state of Mn ions in the NCM811 cathode material [28,29]. The Mn L-edge NEXAFS spectrum of this material also supports this oxidation state (Figure S1). The main energy of the Co ions occurs at 7.721 ± 1 eV, as estimated by the XANES spectrum that coincides with the spectrum of materials with a 3+ oxidation state [30,31]. The Co L-edge NEXAFS spectrum further favors this (Figure S2). The main edge energy value for Ni ions is associated with the 3+ oxidation state, which is characteristic of the NCM811 cathode material and reported by numerous authors [32,33,34]. This oxidation state of Ni ions is also evident from the Ni L-edge NEXAFS spectrum (Figure S3). 

**Figure 3:**
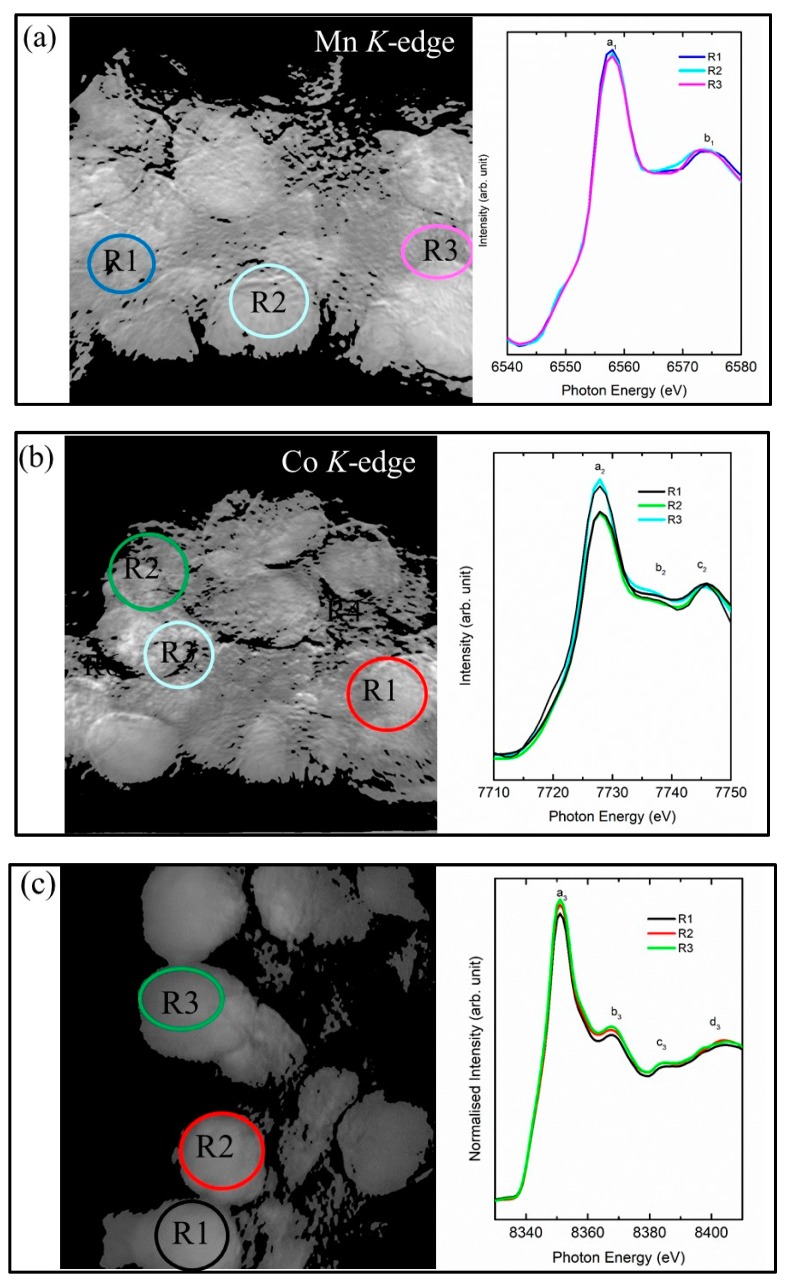
(**a**) Mn *K*-, (**b**) Co *K*-, and (**c**) Ni *K*-edge XANES spectra extracted from each region of interest (ROI), R_1_, R_2_, and R_3_, equivalent to the dimensions of secondary particles.

The secondary particles of cathode materials are generally 10–25 µm; thus the XANES spectrum of the selected region equivalent to the size of the secondary particles is extracted from the specific region of interest (ROI). Figure 3a shows the extracted Mn K-edge spectra of the selected ROI. These spectra exhibit spectral features a_1_ and b_1_ centered at 6.558 ± 1 and 6.573 ± 1 eV, respectively (Figure 3a). These spectral features are clearly visible in the spectra extracted from region R_1_, R_2_, and R_3_ (Figure 3a). This envisages that Mn ions exist at almost the same oxidation state in each region of the cathode materials. Similarly, the Co K-edge spectra for each region R_1_, R_2_, and R_3_ (Figure 3b) exhibit spectral features a_2_, b_2,_ and c_2_ centered at 7.728 ± 1, 7.737 ± 1, and 7.746 ± 1 eV, respectively (Figure 3b). The oxidation state of Ni ions in NMC cathode materials plays an important role during the charging and discharging process. Thus, the Ni K-edge spectra were also measured using XANES-imaging and shown in Figure 3c. The Ni K-edge spectra in the different regions R_1_, R_2_, and R_3_, (Figure 3c) exhibit spectral features a_3,_ b_3_, c_3,_ and d_3_. These spectral features are centered at 8.351 ± 1, 8.368 ± 1, 8.384 ± 1, and 8.404 ± 1 eV, respectively (Figure 3c). Thus, these measurements show that all metal ions exist in an almost matching oxidation state despite the different ROIs. 

**Figure 4:**
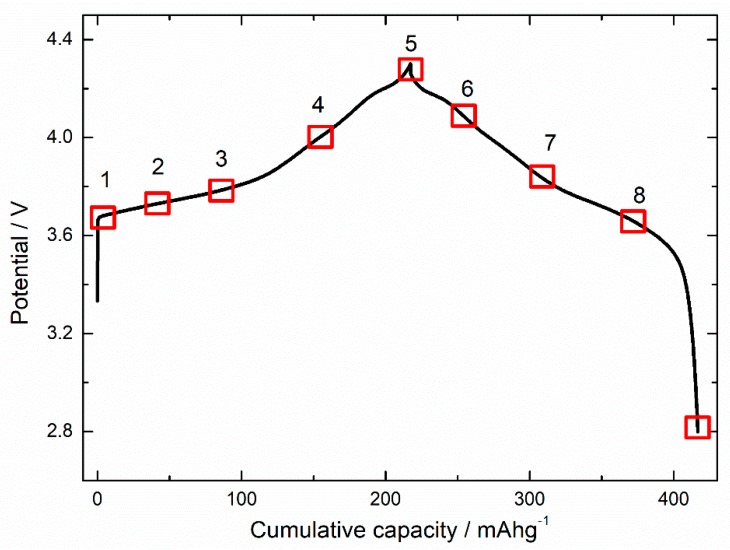
Various states of charging and discharging for NCM811 cathode materials.

The NCM811 cathode material exhibits structure and local electronic structure concurrent with previous reports; therefore, electrochemical testing was performed on this material. Figure 4 shows the charging and discharging curve of the NCM811 cathode material. Various states of charging (SOC) are shown by the numerals, 1, 2, 3, 4, and 5. These numerals denote the charging capacity of 0 (pristine), 40, 100, 160, and 215 mAhg^−^^1^, respectively. The values at the capacity of 247, 302, 357, and 409 mAhg^−^^1^ represent the depth of discharging and are represented by the numerals 6, 7, 8, and 9, respectively. 

**Figure 5:**
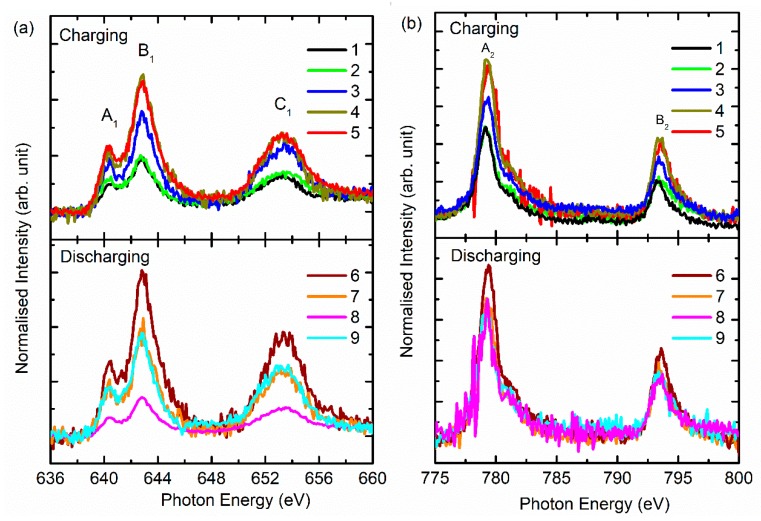
(**a**) Mn *L*-edge and (**b**) Co *L*-edge near-edge X-ray absorption fine structures (NEXAFS) spectra of NCM811 cathode material during charging and discharging.

Figure 5a shows the Mn *L*-edge spectra of NCM811 during charging and discharging. During the charging and discharging process, the spectral features A_1_, B_1_, and C_1_ present in the spectra at various stages of charging and discharging (Figure 5a). The spectral features A_1_, B_1,_ and C_1_ are centered around 640.4 ± 0.4, 642.9 ± 0.4, and 653.3 ± 0.4 eV, respectively, in all states of charging and discharging (i.e., 1–9). The spectral features A_1_ and B_1_ are associated with *L*_3_; however, C_1_ is associated with the *L*_2_-edge. The presence of spectral features that correspond to the *L*_3_ and *L*_2_-edges in the Mn *L*-edge spectra are due to electronic transitions to the 3d levels from the 2p_3/2_ and 2p_1/2_ levels, respectively. The presence of spectral features at an almost identical position (Figure S4) and the lack of change of shape of spectra reveal that Mn ions exist in the same oxidation state during the charging and discharging process. These observations were also obtained from the XANES study of cathode materials during battery operation [32,33].

**Figure 6:**
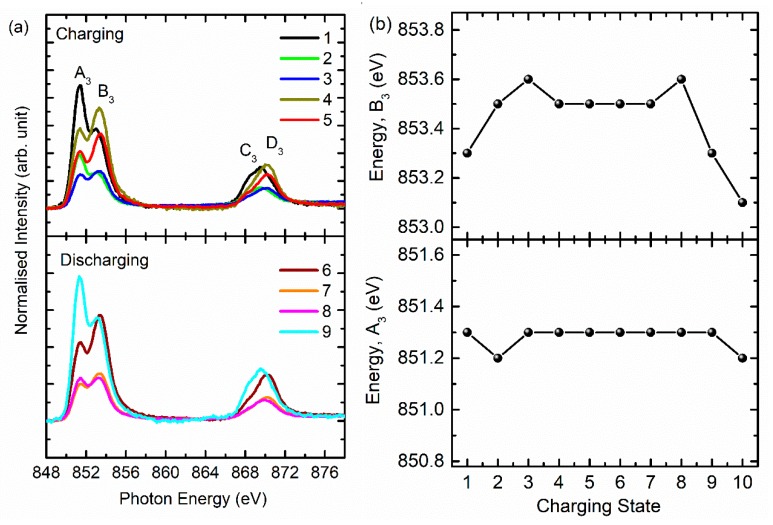
(**a**) Ni *L*-edge spectra and (**b**) energy of spectral features A_3_ and B_3_ of NCM811 cathode materials at stages 1–5 (charging) and 6–9 (discharging).

Figure 5b shows the Co *L*-edge spectra of NCM811 during charging and discharging. During these processes (i.e., 1–9), the spectral features A_2_ and B_2_ appeared in the spectra (Figure 5b). These spectral features were assigned to the *L_3_*- and *L_2_*-edges. Further splitting into *t_2g_* and *e_g_* symmetry state was absent in these spectra. This kind of behavior is observed in Co-based layered oxide cathode materials [35,36]. Spectral features *A_2_* centered at 779.1 ± 0.4 and 793.3 ± 0.4 eV remain at almost the same position during charging and discharging (Figure S5). This demonstrates that during the charging and discharging process, the oxidation state of Co ions does not alter in NCM811 cathode materials. Figure 6a shows the Ni *L*-edge spectra of cathode material during charging (stages 1–5) and discharging (stages 6–9). The spectral features A_3_, B_3_, C_3,_ and D_3_ appear in the spectra for charging and discharging (Figure 6a). These spectral features are centered at 851.4 ± 0.4 (A_3_*)*, 853.3 ± 0.4 (B_3_), 868.4 ± 0.4 (C_3_), and 869.5 ± 0.4 (D_3_) eV in the spectra of the pristine NCM811 cathode. The presence of these spectral features is associated with the presence of Ni ions in an octahedral crystal field. These spectral features represent *t_2g_* (*L_3_*), *e_g_* (*L_3_*), *t_2g_* (*L_3_*), and *e_g_* (*L_3_*) symmetry states. The positions of spectral feature A_3_ remain the same during charging and discharging. The position of spectral feature B_3_ is slightly modified during charging and discharging (Figure 6b); this evinces that the *e_g_* symmetry states are affected dominantly during the charging and discharging state. No role is played by the *t_2g_* states.

**Figure 7:**
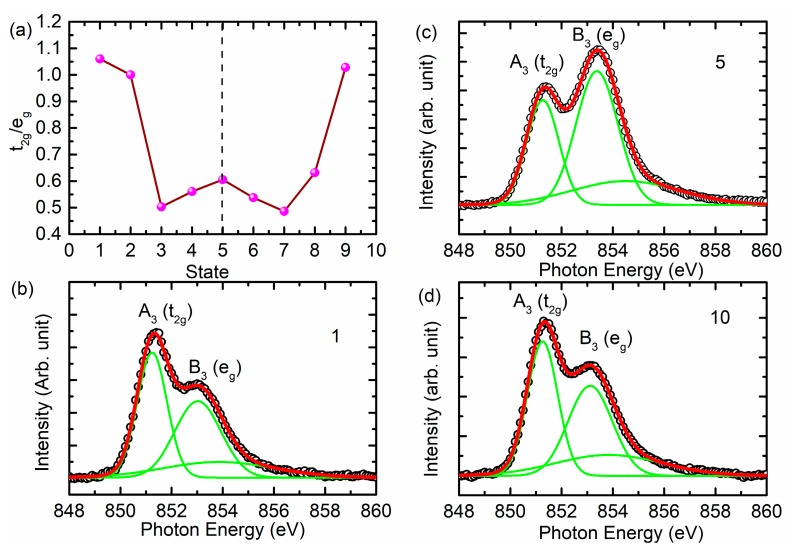
(**a**) The *t_2g_*/*e_g_* ratio estimated from the Ni *L*-edge spectra at various stages of charging and discharging. Here, (**b**–**d**) show the representative de-convoluted spectra for states 1, 5, and 9.

Another interesting change observed in the Ni *L*-edge spectra is the relative change in intensity of spectral features A_3_ and B_3_ associated with the *t_2g_* and *e_g_* symmetry states. The changes are associated with the modification of the oxidation state of Ni ions in the cathode material during charging and discharging [37]. To get deeper insights into this behavior, the area ratio of the spectral features A_3_ and B_3_ was determined (Figure 7a) by de-convoluting the *L_3_*-edge region using the Gaussian function. The de-convolution spectra for states 1, 5, and 9 are shown in Figure 7b–d. The ratio of these spectral features changes alongside the charging and discharging states. The ratio is reduced with charging, indicating transformation to a higher oxidation state. After discharging, the ratio equivalent to the pristine state is observed, indicating that the oxidation state of Ni ions is reversible.

**Figure 8:**
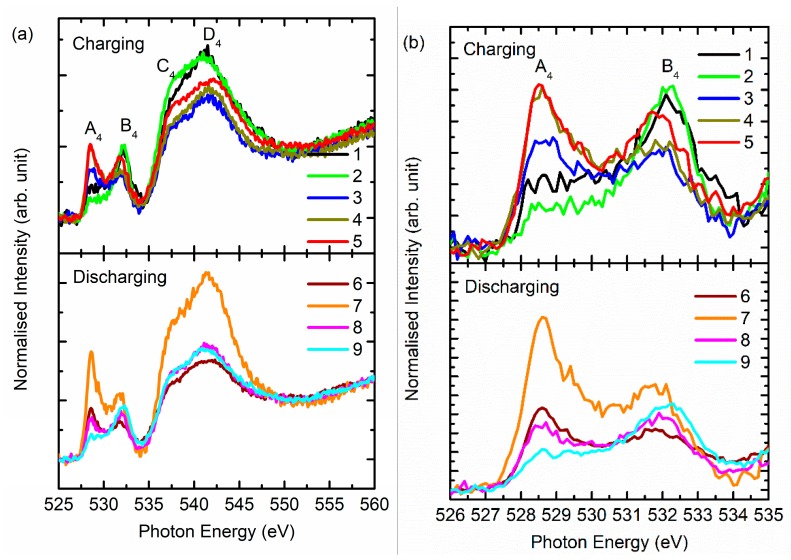
(**a**) O *K*-edge spectra and its (**b**) pre-edge region for the NCM811 cathode material during charging and discharging.

Figure 8 shows the O *K*-edge spectra of the NCM811 cathode during the charging and discharging process. Both the pre-edge and post-edge region of the O *K*-edge spectra reflect the systematic changes during charging and discharging. The pre-edge region of the O *K*-edge spectra exhibits spectral features A_4_ and B_4_ centered around 528.5 and 531.5 eV, respectively, for state 1 (Figure 8b). These spectral features also appear in the spectra for the charging/discharging states 2–9 at almost the same position. The intensities of these features change during charging and discharging.

Features C_4_ and D_4_ appear in the post-edge region of the O *K*-edge spectra (Figure 8a). These spectral features occur at 537.8 ± 0.4 and 541.9 ± 0.4 eV, respectively, for various charging (1–5) and discharging (6–9) states. 

**Figure 9:**
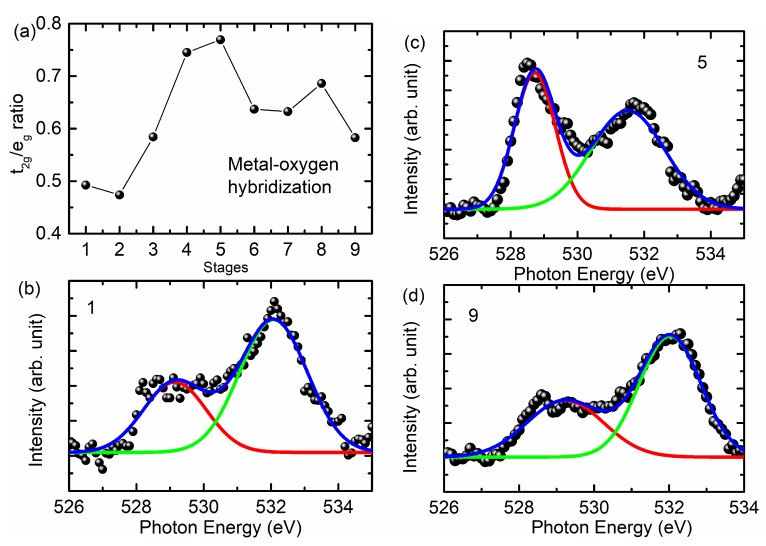
(**a**) The *t_2g_*/*e_g_* ratio estimated from the pre-edge region of the O *K*-edge spectra at various charging and discharging stages. Here, (**b**), (**c**), and (**d**) show the representative de-convoluted spectra for the 1, 5, and 9 states.

Because the pre-edge region of the O *K*-edge spectrum is effective to gather information of metal (3d)–O(2p) hybridization in transition metal oxides [17,18,38]; hence, the intensity ratio of spectral features A_4_ and B_4_ corresponding to *t_2g_* and *e_g_* symmetry state is determined and shown in Figure 9a. Figure 9b–d shows the de-convoluted spectra for states 1, 5, and 9. The intensity ratio is at its maximum for the fully charged cathode and reduces thereafter. Similar behavior is also observed by Tian et al. [39]. This shows the modulation of metal–oxygen hybridization during charging and discharging. The ratio is different from that obtained from the Ni *L*-edge spectra (Figure 7a); this may be due to the influence of these symmetry states from other metal ions such as Mn and Co [39] as well as to the creation of oxygen vacancies during the charging and discharging process [18].

## 4. Conclusions

In conclusion, we have successfully performed soft X-ray spectroscopic measurements during charging and discharging on a well-characterized NCM811 cathode material. These results envisage that oxidation state of Mn and Co ions do not influenced by the charging and discharging process. The oxidation state of Ni ions is affected by charging and discharging; however, in the case of Ni ions, *e_g_* states are dominantly influenced. Although the oxidation state of Ni ions changes, metal–oxygen hybridization during charging and discharging is affected by the presence of other metal ions.

## Figures and Tables

**Table 1:** Structural parameters and reliability factors estimated from Rietveld refinement of the high-resolution powder diffraction (HRPD) pattern of the NMC811 cathode material prior to electrochemical testing.

*a* (Å)/*c* (Å)		2.87402 (2)/14.2018 (1)			
Atom	Site	Wyckoff positions			Occupancy ^*,$^
Li	3a	0	0	0	0.0812 (2)
Ni2	3a	0	0	0	0.0021 (2)
Ni1	3b	0	0	0.5	0.0703 ^†^
Co	3b	0	0	0.5	0.0075 ^†^
Mn	3b	0	0	0.5	0.0032 ^†^
O	6c	0	0	0.2419 (1)	0.166 ^†^
Reliability factors		*R*_p_ = 6.67%, *R*_wp_ = 9.22%, *R*_exp_ = 5.99%, *S* = 1.54			

^*^ The normalized site occupation numbers in % are the following: Li_1_:Ni_2_(97.52:2.48), Ni_1_:Co:Mn (84.48:8.99:3.88), O (100) ^$^ Fixed parameter. ^†^ Occupancy was achieved using the constraints. Li_3a_ + Ni_3a_ = 0.08333.

## Supplementary Materials

The following are available online at https://www.mdpi.com/2079-4991/10/4/759/s1, Figure S1: Mn *L*-edge NEXAFS spectra of NCM811 with reference oxide; Figure S2: Co *L*-edge NEXAFS spectra of NCM811 with reference oxide; Figure S3: Ni *L*-edge NEXAFS spectra of NCM811 with NiO; Figure S4: Positions of spectral features A_1_ and B_1_ of Mn *L*-edge spectra for various states; Figure S5: Positions of spectral features A_2_ and B_2_ of Co *L*-edge spectra for various states.
