# Supplementary material for: Soft X-ray Absorption Spectroscopic Investigation of Li(Ni0.8Co0.1Mn0.1)O2 Cathode Materials
Source: Nanomaterials (Basel). 2020 Apr 15;10(4):759. doi: 10.3390/nano10040759 (PMC7221520; doi:10.3390/nano10040759)
Supplement: Supplementary file 1 [file nanomaterials-10-00759-s001.pdf]

nanomaterials

Supplementary Materials

Soft X-ray Absorption Spectroscopic Investigation of $Li(Ni_{0.8}Co_{0.1}Mn_{0.1})O_2$ Cathode Materials

Jitendra Pal Singh [1], Jae Yeon Park [2], Keun Hwa Chae [3], Docheon Ahn [1,*] and Sangsul Lee [1,*]

1 Pohang Accelerator Laboratory, Pohang University of Science and Technology, Pohang 37673, Korea; jitendra2029@postech.ac.kr

2 Radiation Equipment Research Division, Korea Atomic Energy Research Institute, Jeongup 56212, Korea; jaeyeon@kaeri.re.kr

3 Advanced Analysis Center, Korea Institute of Science and Technology, Seoul 02792, Korea; khchae@kist.re.kr

* Correspondence: sangsul@postech.ac.kr (S.L.); adc4055@postech.ac.kr (D.A.) Tel.: +82-54-279-1569 (S.L.)

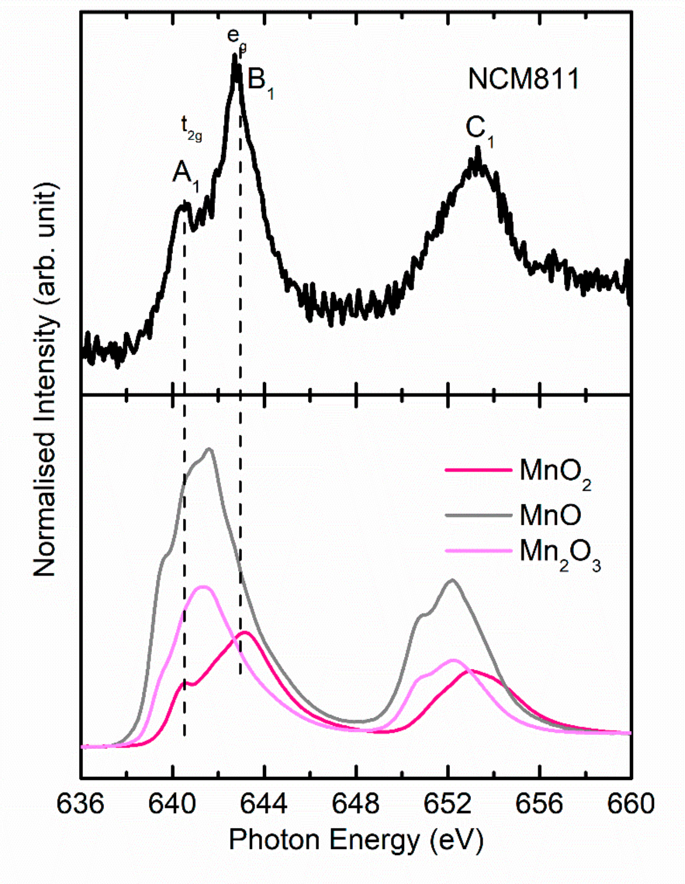

Figure S1: Mn *L*-edge spectrum of NCM811 cathode material along with reference oxide

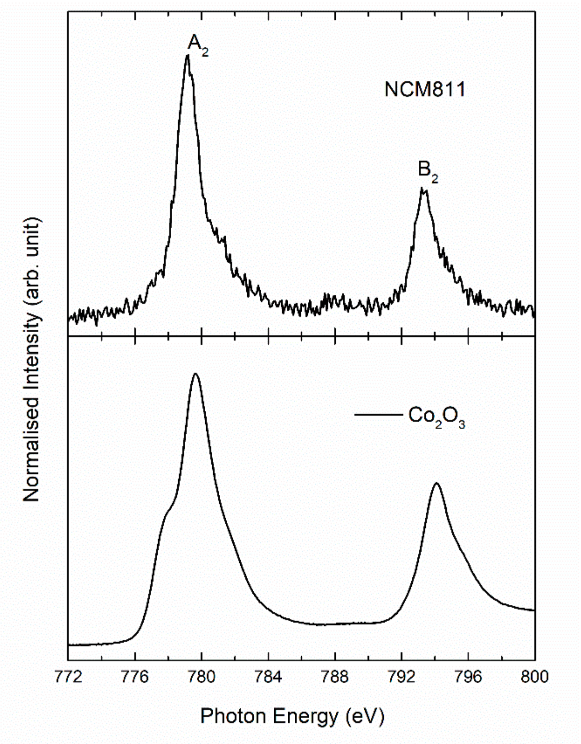

Figure S2: Co *L*-edge spectrum of NCM811 cathode material along with reference oxide.

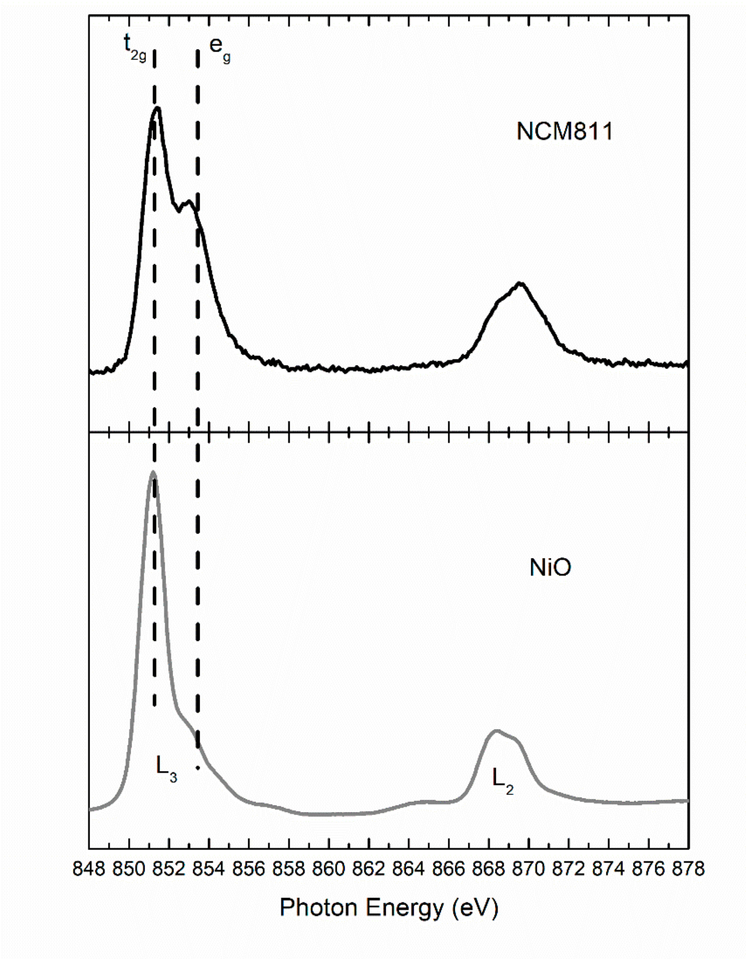

Figure S3: Ni *L*-edge spectrum of NCM811 cathode material along NiO.

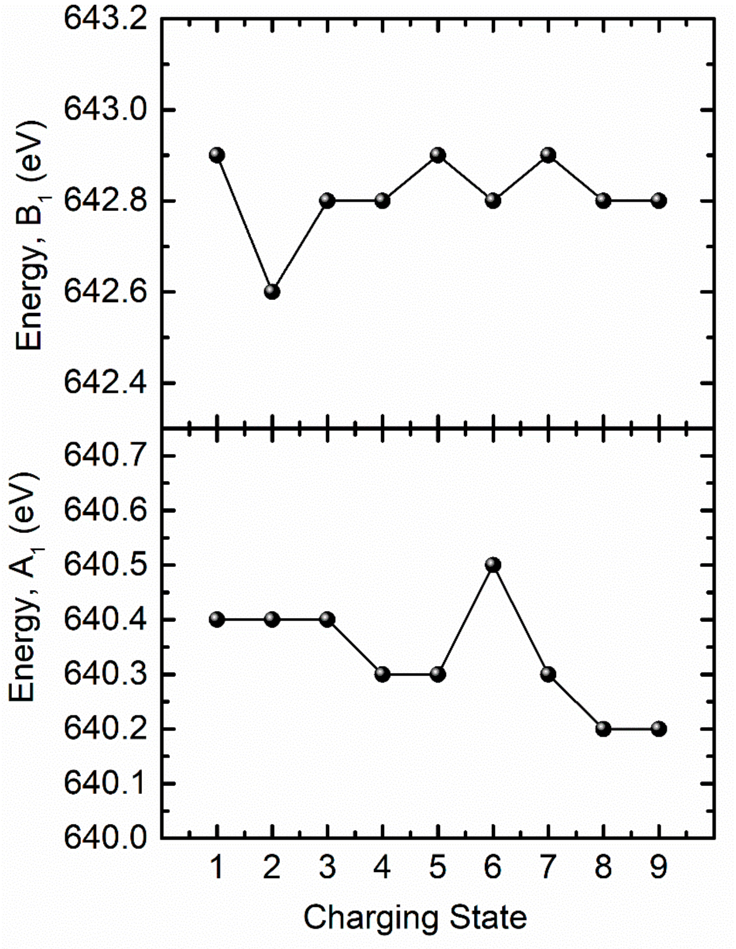

Figure S4: Positions of spectral features A_1, and B_1 of Mn *L*-edge spectra for various states. These spectral features are corresponding to t_{2g} and e_g states.

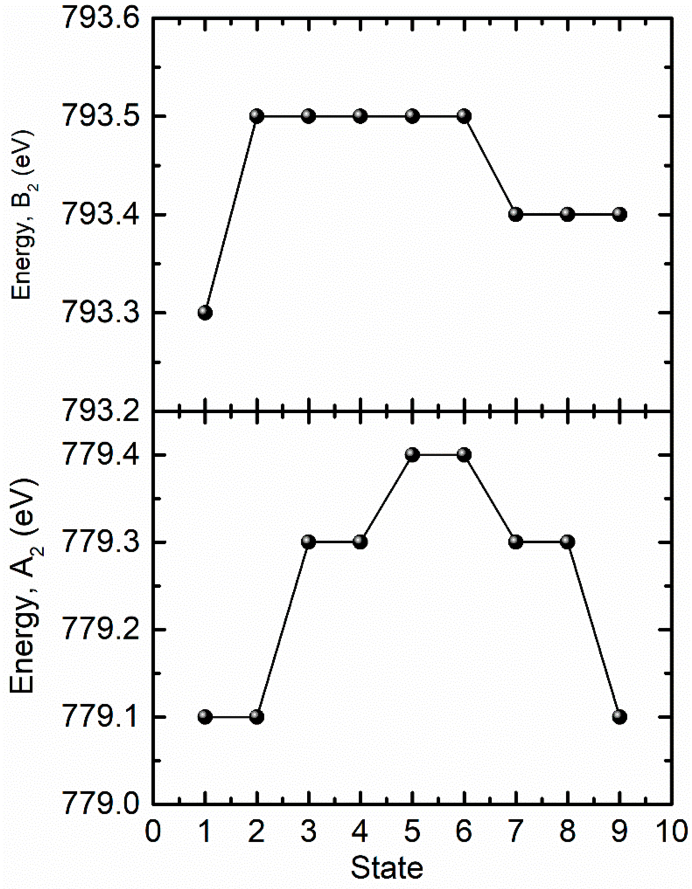

Figure S5: Positions of spectral features A_2, and B_2 of Co *L*-edge spectra for various states.
